# GP assessment of unmet need in a complex multimorbid population using a data-driven and clinical triage system: a prospective cohort study

**DOI:** 10.3399/BJGPO.2023.0078

**Published:** 2023-09-20

**Authors:** Emma Parry, Kamran Ahmed, Simon Evans, Elizabeth Guest, Vijay Klaire, Abdool Koodaruth, Prasadika Labutale, Dawn Matthews, Jonathan Lampitt, Gillian Pickavance, Mona Sidhu, Kate Warren, Baldev Singh

**Affiliations:** 1 School of Medicine, Keele University, Staffordshire, UK; 2 New Cross Hospital, The Royal Wolverhampton NHS Trust, Wolverhampton, UK; 3 Pennfields Medical Centre, Wolverhampton, UK; 4 Coalway Road Practice, Wolverhampton, UK; 5 Warstones Health Centre, Wolverhampton, UK; 6 West Park Surgery, Wolverhampton, UK; 7 Newbridge Surgery, Wolverhampton, UK; 8 Lee Road Medical Practice, Wolverhampton, UK; 9 The City of Wolverhampton Council, Wolverhampton, UK; 10 University of Wolverhampton, School of Medicine and Clinical Practice, Faculty of Science and Engineering, Wolverhampton, UK

**Keywords:** clinical risk, end of life, integrated care networks, multimorbidity, needs analysis, risk stratification, urgent care

## Abstract

**Background:**

Patients with unmet healthcare needs are more likely to access unscheduled care. Identifying these patients through data-driven and clinical risk stratification for active case management in primary care can help address patient need and reduce demand on acute services.

**Aim:**

To determine how a proactive digital healthcare system can be used to undertake comprehensive needs analysis of patients at risk of unplanned admission and mortality.

**Design & setting:**

Prospective cohort study of six general practices in a deprived UK city.

**Method:**

To identify those with unmet needs, the study’s population underwent digitally-driven risk stratification into Escalated and Non-escalated groups using seven risk factors. The Escalated group underwent further stratification using GP clinical assessment into Concern and No concern groups. The Concern group underwent Unmet Needs Analysis (UNA).

**Results:**

From 24 746 patients, 516 (2.1%) were triaged into the Concern group and 164 (0.7%) underwent UNA. These patients were more likely to be older (*t* = 4.69, *P*<0.001), female (X^2^ = 4.46, *P*<0.05), have a Patients At Risk of Re-hospitalisation (PARR) score ≥80 (X^2^ = 4.31, *P*<0.05), be a nursing home resident (X^2^ = 6.75, *P*<0.01), or on an end-of-life (EOL) register (X^2^ = 14.55, *P*<0.001). Following UNA, 143 (87.2%) patients had further review planned or were referred for further input. The majority of patients had four domains of need. In those who GPs would not be surprised if they died within the next few months, *n* = 69 (42.1%) were not on an EOL register.

**Conclusion:**

This study showed how an integrated, patient-centred, digital care system working with GPs can highlight and implement resources to address the escalating care needs of complex individuals.

## How this fits in

Previous attempts to identify patients at risk of unplanned admission and active case management have been unsuccessful. This study presents a novel method of identifying those at risk of unscheduled care use through a data-driven risk stratification model enhanced by GP clinical judgement. The authors present findings on the at-risk cohort identified and the subsequent systematic needs analysis undertaken by the patient’s GP in their role as care coordinator. This study highlights the key domains of need identified and areas of clinical improvement that could be used as a guide to target input from care coordinators.

## Introduction

Unmet needs are the gap between health care required and what is actually provided.^1^ Those with unmet need are more likely to access urgent and unscheduled care due to barriers, such as access and availability, which affects patients’ ability to receive preventative care and chronic disease management.^2^ Unscheduled care use is influenced by factors such as increasing age, lower socioeconomic status, lower educational attainment, presence of multimorbidity and chronic disease, and proximity to unscheduled care.^3^

Outcomes of studies that have analysed the impact of active case management on unplanned admissions and length of stay have been mixed. A cross-sectional survey by Reilly *et al* showed a significant reduction in mean emergency admissions and length of stay in patients with complex health needs.^4^ In contrast, systematic reviews of active case management have not shown statistically significant benefits on unplanned admissions, however they have shown increases in patient satisfaction and self-reported health status.^5,6^ Studies analysing case management are generally of older populations, there is heterogeneity between how individuals are selected for active case management, and these methods may also have inherent weakness affecting the outcome of case management.^5,6^ A large proportion of these studies are also US-based so may not be comparable to the UK, where primary care predominates.^3,6^ Despite this, one important finding is that case management tends to have better outcomes where a multidisciplinary team (MDT) is involved compared to a single case manager.^6^ Promising results have also been found in the field of palliative care, where optimising end-of-life (EOL) care enables patients to have a timely and dignified death.^7^ However, as work in this field has shown one size does not fit all, highlighting the importance of individualised care and service planning.

The King’s Fund describes case management as a targeted community-based tool that takes a proactive approach to those with long-term conditions involving case finding, assessment, care planning, and care coordination.^8^ The overall aim is to reduce unscheduled care consumption, which can be costly and disruptive to patients. Predicting which patients are at high risk of future unscheduled care use is tricky and tools that have been developed for this purpose; for example the Patients At Risk of Re-hospitalisation (PARR) tool^9^ and the Scottish Patients at Risk of Readmission and Admission in Scotland^10^ are hindered by availability, accuracy, and interpretation of key patient data, they are not standardised across healthcare settings, and often lack external validation.^11,12^ Using measures such as previous hospital admissions are susceptible to regression to the mean.^8^

The aim of this current study is to determine how a proactive digital healthcare system can be used to undertake comprehensive complex needs analysis of patients identified at risk of unplanned admission and mortality over the proceeding 12 months.

## Method

### Study design, setting, and practice recruitment

This first phase prospective cohort study was undertaken in the whole population of six general practices in an urban, deprived, multiethnic city in the West Midlands, UK.

All Wolverhampton practices were invited through their Primary Care Networks. Six GP practices volunteered. There were no other selection criteria, nor any financial or other incentive. One GP from each practice was involved in pilot work, study design, and identifying patients for Unmet Needs Analysis (UNA) using a data-driven tool and their own rapid clinical assessment. All six GPs were senior, established practitioners with over 10 years’ experience.

### Data source and variables

The established Wolverhampton Integrated Clinical Data Set links primary care, hospital, and community services data under GDPR regulation.^13^

Demographic variables included age, gender, ethnicity, and the Index of Multiple Deprivation ranked score. Ethnicity data from all sources were reviewed, only unambiguous data were accepted, then recoded into White, South-Asian, Black, Mixed Ethnicity, Chinese, or Unknown.

The comorbidities utilised were the 16 commonest long-term conditions in the population. The variables chosen for risk stratification were based on common variables used in other risk prediction tools and assessed in preliminary work to be linked to emergency activity and mortality.

The seven risk factors used from the Wolverhampton Integrated Clinical Data Set were: ≥3 Accident and Emergency admissions over the prior 12 months not leading to an non-elective admissions (NEA), (to avoid double counting; ≥3 NEAs over the previous 12 months; the 30-day emergency admissions predictor PARR score at a threshold value of 80%;^9^ ≥3 comorbidities; the electronic frailty index (EFI) moderate or severe classification,^14^ nursing home residency, and EOL registration.

### GP rapid clinical assessment and UNA

**Figure 1.**
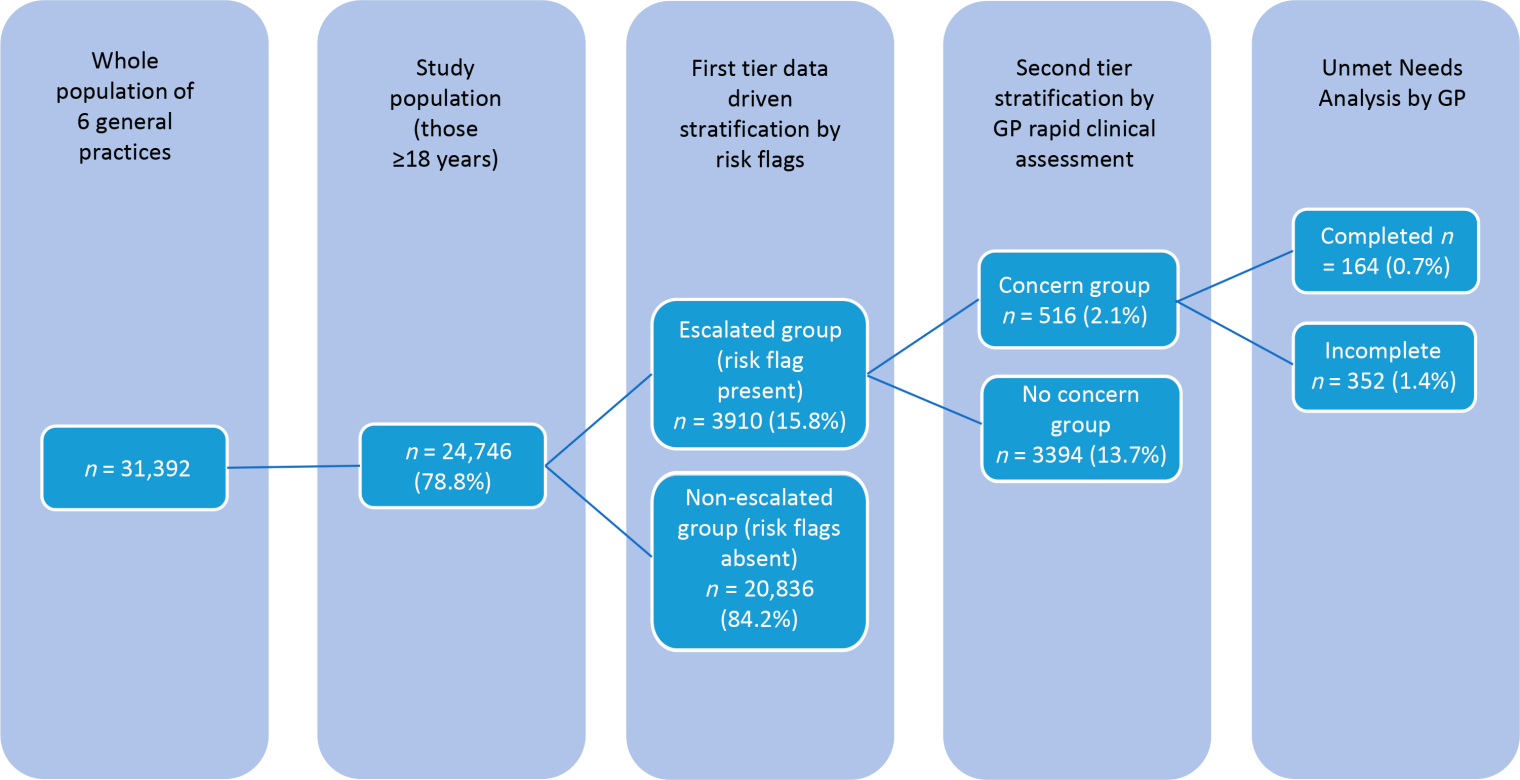
Flow diagram illustrating initial data-driven risk stratification followed by GP rapid clinical assessment using their global clinical judgement.

Patients underwent two-stage stratification in order to identify those with unmet need (Figure 1). The first stage categorised patients into Escalated and Non-escalated groups using data-driven risk stratification based on the seven risk factors described above, which are captured in the Wolverhampton Integrated Clinical Data Set. Those in the Escalated group underwent rapid clinical assessment by their GP and were further triaged into Concern and No concern groups. Stratification into each of these groupings relied on GP clinical judgement and was not defined *a priori*, although discussion with the study GPs agreed that the concept of concern related to whether patients had unmet clinical need, were clinically unstable, might require non-elective emergency care, or were in the last year of life, or would benefit from an MDT process.

Study GPs were asked to undertake an UNA for those in the Concern category. This analysis was captured in a structured electronic form. The form included: the 9-point Rockwood assessment of frailty^15^; the dichotomised UK Gold Standards Framework (GSF) Surprise Question (SQ)^16^ ('Would you be surprised if this patient were to die in the next few months, weeks, days?') to consider those on an end-of-life trajectory; whether the patient was dependent or independent on a 4-point scale; mobility level on a 4-point scale; housebound status, clinician opinion on clinical stability; clinician opinion on likelihood of acute admission in the next 1 month; whether the patient had or required a Do Not Attempt Resuscitation plan; whether patient’s preferred place of death was documented; whether the patient had or required a social care package; and whether the patient had or required a safeguarding review. The clinician then selected actions to be taken, for example adding the patient for MDT review or pharmacy review, and the clinician also indicated when their next GP review should be. All six practices made a coordinated start to the process intending completion within 3 months, with the date of clinical triage as the time stamped anchor point. The project ran and completed to the 90-day post assessment point in October 2021.

### Outcomes

The prospective outcomes of the systematic UNA were the key focus of this paper. Prospectively, 30-day and 90-day event rates were also determined. Non-elective hospital activity was taken as emergency department attendances not leading to a hospital admission or any NEA in the 12 months prior to the date of the study assessments and then in the 90 days thereafter. Mortality was determined from hospital mortality statistics and rolling NHS Strategic Tracing Service checks, thus capturing all known deaths, whether in or out of a hospital setting, that occurred over the prospective 90 days.

The variation between practices in the completion or non-completion of the UNA was analysed independent of demographic and case-mix variation, including nursing home residency, using binary logistic regression.

### Statistical analysis

All data were analysed on IBM SPPS (version 26). The X^2^ test was used for the difference between proportions. Analysis of independent factors with a binary dichotomised dependent variable was by binary logistic regression. Results are presented as the mean ± SD or as numbers with percentages. Statistical significance of all tests applied was taken at *P*<0.05.

## Results

From a base population of 31 392, the adult population aged ≥18 years of 24 746 (78.8%) was selected, among whom: 3910 (15.8%) comprised the Escalated group as they were identified as having one or more of the escalating risk flags; 516 (2.1%) were clinically triaged into the Concern cohort; 164 had a completed UNA, representing 0.7% of the base population or 31.8% of those in the defined Concern group (Figure 1).

Characteristics of the Concern group and of the UNA grouping (complete or incomplete) are given in Table 1. The Concern group were generally older, comorbid, and frail. Those with a completed UNA were more likely to be older (*t* = 4.69, *P*<0.001), female (X^2^ = 4.46, *P*<0.05), have a PARR score ≥80 (X^2^ = 4.31, *P*<0.05), be nursing home resident (X^2^ = 6.75, *P*<0.01) or on the EOL register (X^2^ = 14.55, *P*<0.001), and have an increased 90-day event rate and mortality.

**Table 1.** Demographic characteristics, risk factor variables and 90-day outcomes among those selected by GP assessors to be ‘of Concern‘ who did or did not have an UNA undertaken

	Concern group*n* = 516 (%)	UNA incomplete*n* = 352 (%)	UNA complete*n* = 164 (%)	
**Patient characteristics**				
Age, years	78.5 ±13.2	76.7 ±13.7	82.5 ±11.2	*t* = 4.69, *P*<0.001
Female gender	286 (55.4)	184 (52.3)	102 (62.2)	X^2^ = 4.46, *P*<0.05
White ethnicity	359 (69.6)	247 (70.2)	112 (68.3)	X^2^ = 0.27, ns
Index of Multiple Deprivation score	26.9 ±14.7	27.7 ±15.1	25.3 ±14.2	*t* = 1.67, ns
**Risk factors**				
≥3 A/E admissions over preceding 12 months	45 (8.7)	28 (8.0)	17 (10.4)	X^2^ = 0.82, ns
≥3 NEA over preceding 12 months	42 (8.1)	26 (7.4)	16 (9.8)	X^2^ = 0.84, ns
PARR score ≥ 80	57 (11.0)	32 (9.1)	25 (15.2)	X^2^ = 4.31, *P*<0.05
≥3 Comorbidities	438 (84.9)	300 (85.2)	138 (84.1)	X^2^ = 0.10, ns
EFI (moderate or severe)	334 (64.7)	223 (63.4)	111 (67.7)	X^2^ = 0.92, ns
Nursing home resident	79 (15.3)	44 (12.5)	35 (21.3)	X^2^ = 6.75, *P*<0.01
On the End-of-Life register	136 (26.4)	75 (21.3)	61 (37.2)	X^2^ = 14.55, *P*<0.001
**90-day outcomes**				
Any A/E attendance	119 (23.1)	74 (21.0)	45 (27.4)	X^2^ = 2.60, ns
Any NEA	86 (16.7)	53 (15.1)	33 (20.1)	X^2^ = 2.07, ns
Mortality	42 (8.1)	22 (6.3)	20 (12.2)	X^2^ = 5.29, *P*<0.05
Any event	146 (28.3)	88 (25.0)	58 (35.4)	X^2^ = 5.93, *P*<0.02


Results are the mean ± the SD or numbers with percentages.

A/E = Accident/Emergency; EFI = electronic frailty index; NEA = non-elective admission; PARR = Patients at risk of re-hospitalisation; UNA = unmet needs analysis;ns = non-significant;

There were between-practice differences in several regards: the proportion of patients with defined risk factors (X^2^ = 182.6, *P*<0.001; range 10.3%–20.6%); from among those that were risk escalated, the proportion clinically triaged into the Concern cohort (X^2^ = 387.3, *P*<0.001; 2.5%–27.6%); the proportion in whom UNA was undertaken from within the Concern cohort (X^2^ = 5.6, *P*<0.001; 0–85.2% [mean 35.0%]). Statistical analysis of the variation between practices in completing the UNA was highly significant (*P*<0.001) independent of any differences in demographic and clinical variables (X^2^ = 82.8, *P*<0.001, *r*2 = 0.21).

A classification of the outcomes of the UNA are given in Table 2. These patients were clinically complex, the majority were frail, answer to the GSF SQ was ‘no’ in 76.2%. Of those where the answer to the SQ was ‘no’, 63.2% required place of death wishes to be coded, but it was not recorded, and 29.6% required an advance care plan, but this was not recorded. There was no indication that the prediction of possible or probable acute admissions in UNA identified those who went on to have acute admissions as 20.3% (*n* = 24) of those predicted to need admission were admitted. Conversely, of those who were not predicted to be admitted 19.6% (*n* = 9) were (X^2^ = 0.01, not significant).

**Table 2.** A reclassification of the separate needs arising out of the UNA tool into nine categorised domains (*N* = 164)

Aiding understanding of clinical complexity	*n (%)*				Answered 'no' to the Surprise Question (*n* = 125)
Dependency (partial *n* = 76, full *n* = 49)	125 (76.2)				
Immobility (partial *n* = 64, full *n* = 42)	106 (64.6)				
Housebound	80 (48.8)				
Frailty (Rockwood; moderate 41, severe 83)	124 (75.6)				
**Urgent care**					
Imminent acute admission within 30 days (possible *n* = 103, probable *n* = 15)	118 (72.0)				
**End of life care**					
GSF surprise question = No	125 (76.2)				
GSF prognosis (not given *n* = 18/months *n* = 102/weeks *n* = 4/days *n* = 1)	107 (65.2)				(85.6% of 125)
Join directly to EOL pathway	15 (9.1)				(12.0% of 125)
Do not attempt resuscitation required	74 (45.1)				(59.2% of 125)
Advanced care plan required	37 (22.5)				(29.6% of 125)
Place of death wishes record required	79 (48.1)				(63.2% of 125)
**Direct patient contact**					
Telephone the patient	37 (22.6)				
Arrange appointment for the patient	32 (19.5)				
Any planned direct patient contact (of the above either 62, both 26)	43 (26.2)				
**Planned follow-up by GP**					
Systematic re-review (3, 6, 12 months; *n* = 34/57/12)	103 (62.8)				
**Referral within the primary care Integrated Care Network**					
Practice nurse review	29 (17.7)				
Community team review	19 (11.6)				
Care package required	18 (11.0)				
Pharmacist review	4 (2.4)				
Social services review	3 (1.8)				
Safeguarding review	1 (0.6)				
**Formal MDT**					
Add to formal MDT	32 (19.5)				
**Referral to specialist services**					
Any referral	50 (30.5)				
**Other**					
Other miscellaneous actions	39 (23.8)				

**Figure 2.**
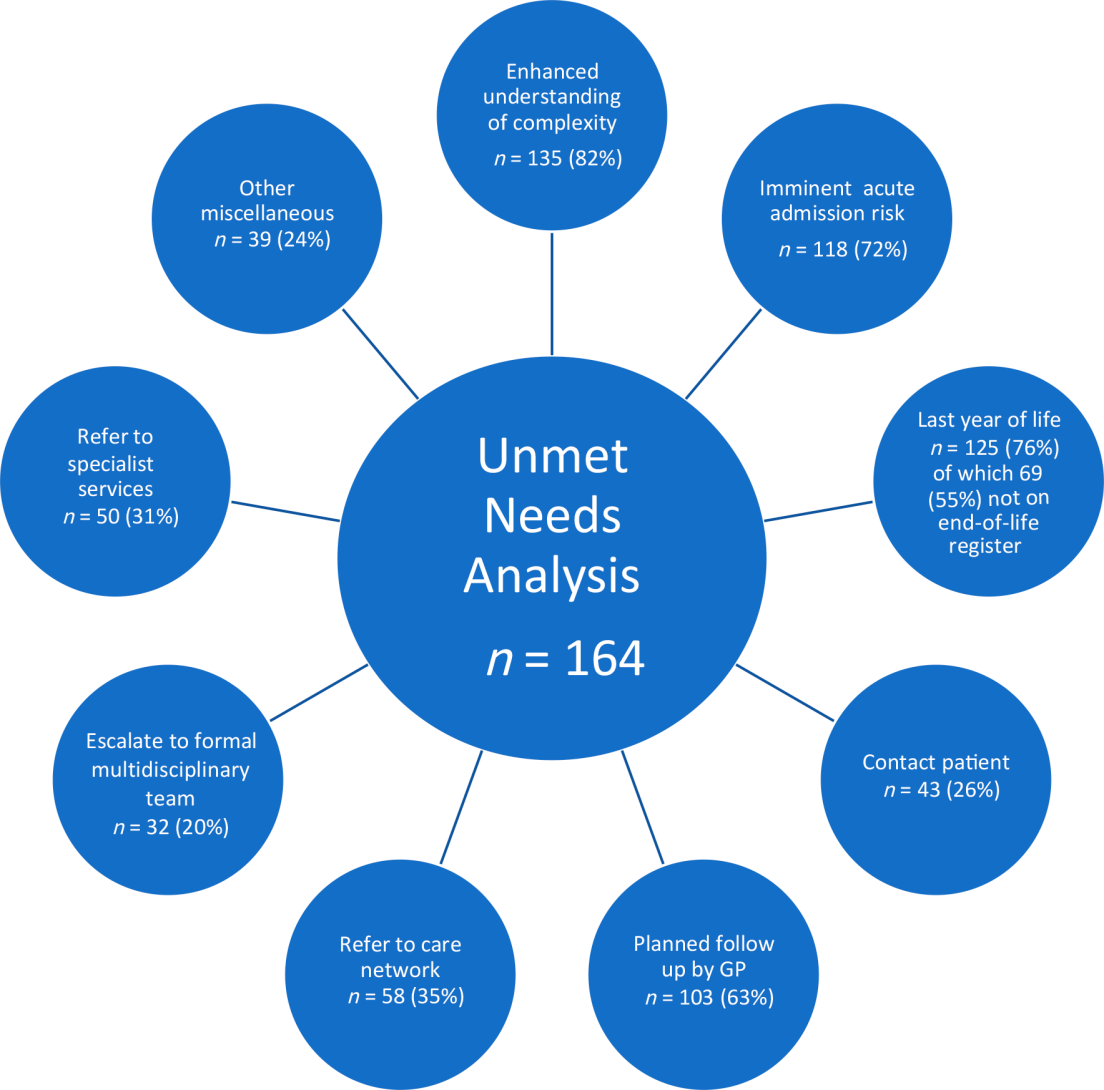
The global classification domains of the individual items of unmet care needs analysis (see Table 2).

EOL = End of life; GSF = Gold standard framework; MDT = Multidisciplinary team; UNA = Unmet needs analysis;

Following UNA 143 (87.2%) of patients had a further review planned or were referred for further input; direct immediate contact was stipulated in 26.2% (telephone or face-to-face); 19.5% were referred to MDT; 17.7% were referred for practice nurse review; smaller numbers were referred for community team review, pharmacist review, social services review or safeguarding review; there was a planned elective review with a GP in 62.8% of cases; 30.5% were referred onwards to specialist services, although this was not further classified (Figure 2). The majority of patients had 4 domains of need (**Supplement 1**).

The classification of frailty differed by the two methods used (X^2^ = 39.1, *P*<0.001, Table 3), with clinician assessment by Rockwood scoring identifying an additional 24 patients (14.6%) with moderate or severe frailty, noting a declassification in 11 (6.7%), and concordance in 129 (78.7%) of patients.

**Table 3.** A comparison of the classification of moderate or severe frailty either by EFI or Rockwood (X^2^ = 39.1, *P*<0.001)

	Rockwood	
	No (%)	Yes (%)
**EFI No**	29 (17.6)	24 (14.6)
**EFI Yes**	11 (6.7)	100 (61.0)

EFI = electronic frailty index;

Table 4 shows the relationship between the EOL assessment undertaken by answering the GSF SQ in UNA to actual recorded status in the EOL register (X^2^ = 13.0, *P*<0.001) and this shows concordance in only 90 (54.8%), while 69 (42.1%) with an SQ outcome ‘No’ were not on the EOL register, and 5 (3.0%) with a SQ outcome ‘Yes’ were. Of note, the GSF SQ ‘No’ classification by GPs encompassed 18 of 20 (90.0%) deaths at 90 days where the UNA was undertaken but this prediction did not attain statistical significance (X^2^ = 3.35, *P* = 0.34), possibly reflecting a type-two statistical error since statistical power calculation for 90-day mortality showed a required total population size of 688 (versus 164 complete) to show significance at the 1% level with a power of 80%.

**Table 4.** The relationship between potential end of life status assessed by the GSF SQ, 'Would you be surprised if this patient were to die in the next few months, weeks, days?', to the recorded status in the EOL register (X^2^ = 13.0, *P*<0.001)

	GSF SQ	
	Yes (%)	No (%)
**EOL register No**	34 (20.7)	69 (42.1)
**EOL register Yes**	5 (3.0)	56 (34.1)

EOL = end of life; GSF = Gold Standard Framework; SQ = Surprise Question;

## Discussion

### Summary

This study identified a cohort of patients, through digital and clinical risk stratification who had unmet healthcare needs and were at risk of acute hospital admission. These patients were more likely to be older, female, be a nursing home resident, be on an EOL register, increased likelihood of a PARR score ≥80, and had increased 90-day event rates. Through the UNA, GPs were able to determine the appropriate care needs of their complex patients and highlighted areas for improvement. In those who had a UNA, GPs rated over three-quarters of patients as ‘no’ to the GSF SQ, however of these only 42.07% were not on an EOL register.

### Strengths and limitations

Limitations include the small scale of the study. The assessors were research-active, senior GPs and therefore may not be representative of the wider GP population. There was significant variation between practices in the proportion of UNA that took place. The authors did not capture any causes of this variation but anecdotal feedback from study GPs included resource and time constraints as practices emerged from the COVID-19 pandemic. Care outcomes were not studied so this study could not report on the impact of the needs analysis. Strengths include collection and utilisation of integrated data from primary, community, and secondary care sources to identify cohorts with unmet healthcare needs who would benefit from further input.

### Comparison with existing literature

Similar to this study's findings, other studies have found that those who are older, more socially deprived, have comorbidity, or are EOL are at increased risk of unscheduled care use.^17,18^ Illness trajectories for those with terminal conditions are well established and generally follow one of three well-known patterns: steady progression with obvious terminal phase; gradual decline with episodes of acute deterioration with some recovery; and prolonged gradual decline (similar to frail older people).^7^ With this in mind, it is surprising that a large proportion of this study's cohort (42.1%) who were expected to not be alive in the next 12 months were not on an EOL register. This contrasts with the 2009 National Snapshot in Primary Care where 4487 deaths were analysed from 502 GP practices, between February and March 2009, finding that 15% of deaths were thought to be predictable, but patients were not on an EOL register.^19^ The authors’ system identified patients earlier allowing for anticipatory care planning. Identifying and flagging those who are approaching EOL is important for identifying unmet needs, providing quality holistic care, supporting carers, and providing information as early as possible so that patients can make informed decisions about their future care and death.^20^

Increased input in those with unmet needs has shown some benefits. A study of patients with cancer who had increased contact with community nurses in the last 3 months of life were shown to have less unscheduled care use.^21^ A systematic review, performed in the US, exploring the impact of intensive primary care intervention on mostly older patients with functional limitation, showed mixed results; there was some evidence that input from an MDT led to fewer hospital admissions but no impact on emergency department admissions or mortality. However, evidence was generally weak and there was heterogeneity in the different cohorts and how they were selected for intense intervention^22^

### Implications for research and practice

This study improved our understanding of complexity and frailty in the primary care population studied and it highlighted those in need of specific input such as direct contact, care packages, those needing specific onward referral, or who would benefit from an MDT process. The authors believe that better identification of those at risk of unscheduled care is attainable through a novel data-driven model, which is enhanced by the clinical judgement of a patient’s GP.

Based on this pilot study, the authors estimate that practices with an average list size of 10 000 will have an initial 100 (1%) patients who will require detailed UNA. Feedback from study GPs stated the time to complete rapid clinical assessment and UNA ranged from 5–10 minutes for patients well-known to them to up to 30 minutes for more complex patients who were less known to them. Despite this, once the initial phase dealing with the base prevalence of the requirements was completed, GPs can keep abreast of the incidence of newly identified at risk patients, at time points convenient to them (for example daily, weekly, or monthly). The UNA does not need to be completed by GPs, but could be done by suitably trained members of the primary care team.

Once this at-risk cohort is identified, the authors have demonstrated a systematic process for identifying unmet care needs and the common domains of potential need which will help with resource planning.

The next phase of this project is to analyse the impact of the UNA and subsequent intervention to explore whether this has an impact on unscheduled care use and mortality in the population, including a cost–benefit analysis. The authors will also determine the reasons for variation in the completion of the UNA, with a particular focus on time, workload, training requirements, and resource constraints.

This study showed how an integrated, patient-centred, digital care system working with GPs can highlight and implement resources to address the escalating care needs of complex, comorbid individuals in a population.

## Availability of data and materials

The datasets used and/or analysed during the current study are available from the corresponding author on reasonable request.
